# An Address to the Graduates of the Ohio College of Dental Surgery, Session of 1858–9

**Published:** 1859-03

**Authors:** W. W. Allport

**Affiliations:** Chicago


### EXTRACTS.

*An Address to the Graduates of the Ohio College of Dental Surgery, session of* 1858-9, *by* ***W. W.*** *Allport, D.D.S., of Chicago.*

The invitation extended to Dr. Allport, to address the graduating class of the Ohio College of Dental Surgery, was a marked compliment to one of our citizens, and a tribute to his skill and character. He is well known, and his skill is appreciated here. It is gratifying to know that his position is no less recognized abroad. That which we did not know, however, and what the Doctor has taken care to show us, in this address, is the fact that his pen is as sharp as his instruments, and comes as directly to the point. In proof and illustration, we should be pleased to present the whole of this address to our readers, but for want of space must be content with one or two passages, not inapplicable to the medical, as well as to the dental profession.

"It is necessary that you should have a manly independence, and to feel this you must be out of debt. If the kindness of friends, or the forbearance of your teachers, has furnished you with the means of education, or assistance, to enable you to enter upon the discharge of your professional duties, you ought to repay them from your very first earnings. No man has a free will and cheerful heart, who goes to his work the slave of debt. It fetters, at every step of life, and leaves the door open for continual misunderstanding, and may sever the closest friendship. A prompt paymaster must be a good collector. Success in any business depends upon a careful attention to bills payable and bills receivable. You will also find that work which is paid for will fit better, and last longer, than work unpaid for. With some patients, few things stop the uneasiness of a newly filled tooth, or remedy the defects in a new set of teeth, or add to his reputation, like the honest payment of the dentist's bill. Few callings give greater room for misunderstanding than our own, but you will find that honest work for honest people will give but little trouble. * * * *

"You should husband well your time, and remember that life and reputation are made up of little things; that every act adds to or detracts from an honored name. You can not expect to gather figs of thorns, or grapes of thistles. Character is not a thing of chance, nor skill the reward of ignorance or idleness."

